# Formability of the 5754-Aluminum Alloy Deformed by a Modified Repetitive Corrugation and Straightening Process

**DOI:** 10.3390/ma13030633

**Published:** 2020-01-31

**Authors:** Marco Ezequiel, Sergio Elizalde, José-María Cabrera, Josep Picas, Ignacio A. Figueroa, Ismeli Alfonso, Gonzalo Gonzalez

**Affiliations:** 1 Instituto de Investigaciones en Materiales, Universidad Nacional Autónoma de México, Circuito Exterior S/N, Cd. Universitaria, A.P. 70-360, Coyoacán, Ciudad de Mexico 04360, Mexico; marcoeze13@gmail.com (M.E.); iafigueroa@unam.mx (I.A.F.); 2 Departamento de Ciencia e Ingeniería de Materiales, EEBE-Universitat Politècnica de Catalunya, c/Eduard Maristany 10-14, 08019 Barcelona, Spain; sergioelizalde85@gmail.com (S.E.); jose.maria.cabrera@upc.edu (J.-M.C.); 3 Instituto de Investigaciones en Metalurgia y Materiales, Universidad Michoacana de San Nicolás de Hidalgo, Edificio U, Av. Francisco Múgica s/n, CU, Morelia 58000, Michoacán, Mexico; 4 Light Alloys and Surface Treatments Design Centre, Department of Materials Science and Metallurgy, Universitat Politècnica de Catalunya, Rambla Exposició 24, 08800 Vilanova i la Geltrú, Spain; josep.picas@upc.edu; 5 Instituto de Investigaciones en Materiales, Unidad Morelia, Universidad Nacional Autónoma de México, Campus Morelia UNAM. Antigua Carretera a Pátzcuaro No. 8701, Col. Ex-Hacienda de San José de la Huerta, Morelia C.P. 58190, Michoacán, Mexico; ialfonso@unam.mx

**Keywords:** aluminum alloys, texture, strain-rate sensitivity, RCS, forming capacity

## Abstract

Sheets of 5754-aluminum alloy processed by a modified repetitive corrugation and straightening (RCS) process were tested in order to measure their formability. For this purpose, forming limit curves were derived. They showed that the material forming capacity decreased after being processed by RCS. However, they kept good formability in the initial stages of the RCS process. The formability study was complemented with microstructural analysis (derivation of texture) and mechanical tests to obtain the strain-rate sensitivity. The texture analysis was done by employing X-ray diffraction, obtaining pole figures, and the orientation distribution function. It was noticed that the initial texture was conserved after successive RCS passes, but the intensity dropped. RCS process did not induce β-fiber, contrary to common deformation process. The strain-rate sensitivity coefficient was measured through tensile tests at different temperatures and strain rates; the coefficient of the samples processed after one and two passes were still relatively high, indicating the capacity to delay necking, in agreement with the good formability observed in the initial passes of the RCS process.

## 1. Introduction

Aluminum alloys are widely used in industry due to their specific strength and forming capacity. Specifically, aluminum sheets are one of the most required products for deep drawing applications. The AA5xxx series combine their strain hardening capacity with corrosion resistance, and acceptable forming capacity [1,2]. The effect of cold working in AA5xxx sheets have been widely studied [3,4]. The effect of plastic deformation methods—such as equal-channel angular pressing (ECAP), accumulative roll bonding (ARB), high pressure torsion (HPT), and constrained groove pressing (CGP), among others—have been reported for aluminum sheets [5,6,7,8]. However, to date, there are no reports of Al-5754 alloy processed by the so-called Repetitive Corrugation and Straightening (RCS) process, despite showing better forming capacity in comparison with other alloys from the same 5xxx series, and having applications on the automotive industry, e.g., on automotive panels [9]. On the other hand, it has been observed all above-mentioned processes often lead to a substantial detriment in the forming capacity of the materials.

The forming capacity in metal or alloy sheets can be assessed in different ways, being one of the most popular the derivation of the Forming Limit Diagrams (FLD), [10,11]. This technique gives reliable information on the fracture limit for the most widely used or investigated deformation states. It is evident that the crystallographic texture affects the metallic formability and, in this sense, several attempts have been made in the literature to find out the correlation between texture components on the forming capacity of sheet samples, in metals in general, and in aluminum in particular [12,13].

In FCC metals the stacking fault energy determines whether the plastic deformation occurs by slip and/or twinning. In the case of aluminum, slip on the systems {111} <110> is the predominant deformation mechanism [14]. In this sense, the beta fiber that contains the cube, S and brass components is widely known to be formed on rolled aluminum samples [15,16]. Nevertheless, a lack of systematic analysis has led to inconsistent and even contradictory conclusions regarding these texture components and their impact on forming capacity [17]. For instance, Yoshida et al. [18] claimed that cube texture is beneficial for forming capacity, whereas other authors [19,20] reported that such texture is detrimental. Similarly, for the brass component [17,21], it has been reported the enhancement of the formability in contrast with that reported that both brass and S components reduce the forming capacity [18]. However, it has been assessed that, in order to correlate the forming capacity with the crystallographic texture, the FLD needs to be obtained [13].

On many occasions, especially under limited formability conditions, deformation is carried out at high temperatures. Under this condition, the strain-rate sensitivity coefficient can be used as a formability measure. This coefficient can be used as a practical value of the material ability to avoid necking, and consequently, the capacity to keep homogeneous deformation and delay fracture [22,23]. Moreover, this value has been related to changes in specific regions of the FLD curves [20]. Specifically, high strain-rate sensitivity has been found on modified AA5xxx [24,25].

In consequence, the objective of this work is to study the impact of a modified RCS process (a variant of severe plastic deformation method) on the forming capacity of 5754-aluminum alloy sheets. In order to achieve this objective, FLD, macrotexture analysis, and strain-rate sensitivity measurements were carried out.

## 2. Materials and Methods

### Material

Commercial sheets of 5754-aluminum alloy with H111 temper were used as starting material, its nominal composition is shown in Table 1.

## 3. Modified RCS

**Figure 1:**
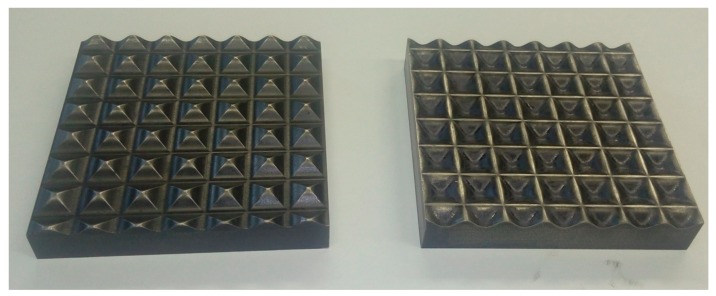
New die design used for the RCS process.

Sheets of 5754-aluminum alloy, with dimensions of 12 × 12 × 1 mm, were processed by a new RCS die design, with the aim of inducing heterogeneous deformation over the samples. The new die is shown in Figure 1, being designed with the sinusoidal function

(1) $$f\left(x\right)=2\sin\left(\frac{\pi }{8}x\right)$$

where *f*(*x*) is the teeth height and *x* is the horizontal coordinate, both in mm.

Details of the process were discussed in a previous work [26]. One RCS pass is defined as one corrugation and straightening press followed by a 180° sheet rotation and another corrugation and straightening press. This was performed in order to generate highly deformed zones surrounded by areas with low deformation. With the aim of studying the forming capacity changes, two RCS passes were carried out. The samples here studied are subsequently referred as 0P, 1P, and 2P, indicating the number of RCS passes.

After the RCS, some mechanical properties of interest were also evaluated. Yield strength and elongation were measured at room temperature on standard tensile specimens (gage length of 50 mm), using an INSTRON 5500R universal testing machine at a strain rate of 3 × 10^−4^ s^−1^, three specimens were tested and the average values were plotted. Besides, the hardness values were monitored using a Shimadzu HMV-G, applying 100 g for 10 s (HV_0.1_).

## 4. Forming Limit Diagrams

**Figure 2:**
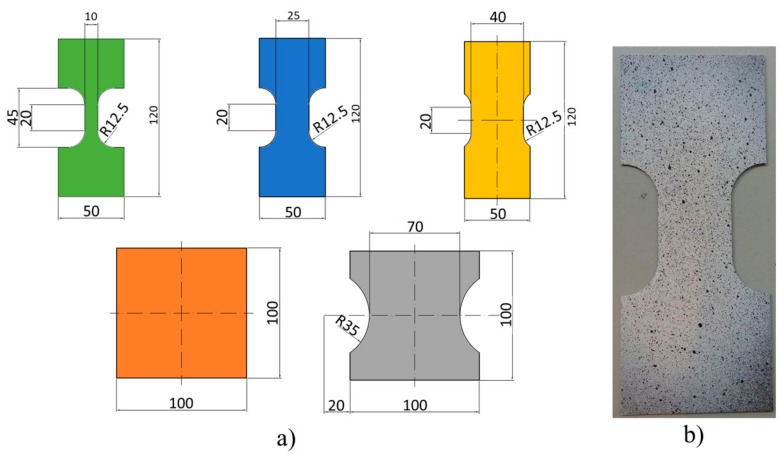
(**a**) Geometries for the Nakajima (all dimensions are in mm) and (**b**) sample painted with the stochastic pattern, intended to follow the deformation during the tests.

In order to trace the forming limit diagrams (FLD), Nakajima tests were carried out at room temperature. For this purpose, samples with the geometries and dimensions shown in Figure 2 were machined. In order to avoid friction effects, Teflon was used in a Teflon-PVC-Teflon configuration placed between the sample and the punch.

**Figure 3:**
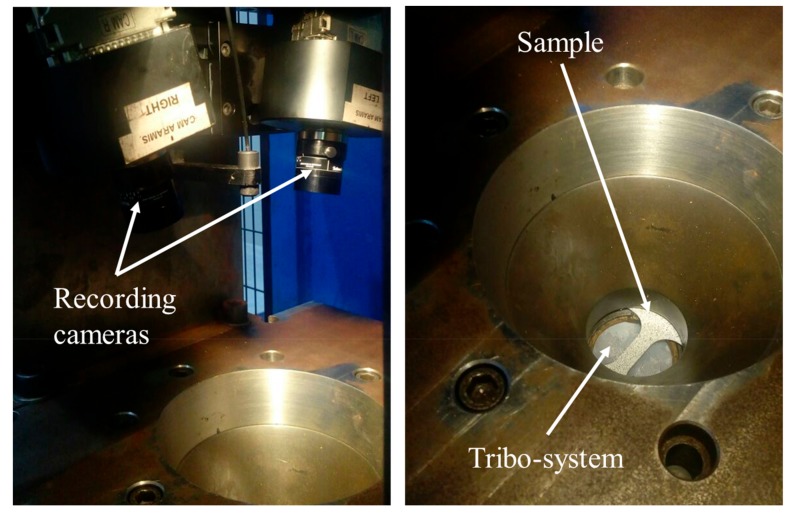
Setup for the Nakajima test with two recording cameras, the sample and the tribo-system.

The samples were painted with a stochastic pattern in order to have a contrast for the strain measurements using a digital image correlation system (Figure 2). According to ISO 12004-2:2008, the test was carried out with a loading speed of 1 mm/s, stopping the test when the fracture was detected. The sheets were held by the press during the Nakajima tests. Two recording cameras were used for obtaining images during the test; the deformation states were measured through the image analyzer software ‘ARAMIS’. The experimental setup is shown in Figure 3. To determine the deformation across the sheet, a comparison of the pattern was made before and after the test. The test was performed using three samples for each geometry, measuring the strain of three sections for each sample to have reliable statistics. The sections analyzed were defined perpendicular to the fracture line.

The forming limit curves (FLC), for the samples before and after both RCS passes, were drawn using the obtained values for the minor and major strains at fracture. The FLCs were compared with FLC from A5083 and A6111-T4 alloys that are widely used in industrial applications, and that requires high forming capacity.

## 5. Macrotexture Analysis

Pole Figures of the crystallographic (111), (200), and (220) planes were obtained for the different RCS passes, using a Rigaku Ultima IV X-ray diffractometer, with a wavelength of Cu-Kα, equipped with crossbeam optics, in mode “In-Plane”. MATLAB software with the MTEX toolbox [27] was implemented to calculate the orientation distribution function for each sample in order to study the possible changes in texture and volume fraction.

## 6. Strain-Rate Sensitivity

Tensile tests were conducted on samples processed by RCS at different temperatures and strain rates. Standard tensile specimens (gage length of 50 mm) were tested at temperatures ranging from 150 to 400 °C, and at strain rates between 3 × 10^−5^ s^−1^ and 0.3 s^−1^. The main goal of these tests was to calculate the strain-rate sensitivity coefficient *m* according to the equation

(2) $$\sigma =K{\dot{\varepsilon }}^{m}$$

where *σ* is the stress, *K* is a constant, $\dot{\varepsilon }$ is the strain rate and, *m* is the strain rate sensitivity coefficient.

The coefficient *m* is directly correlated to the forming capacity of the material at the test temperature [22]. For the calculation, at least two tensile tests were carried out at the same temperature and strain rate. The maximum stress values were recorded with the corresponding strain rate. These points were plotted on a logarithmic scale; then, a linear regression was applied. The slope on that plot corresponds to the *m* coefficient.

## 7. Results and Discussion

**Figure 4:**
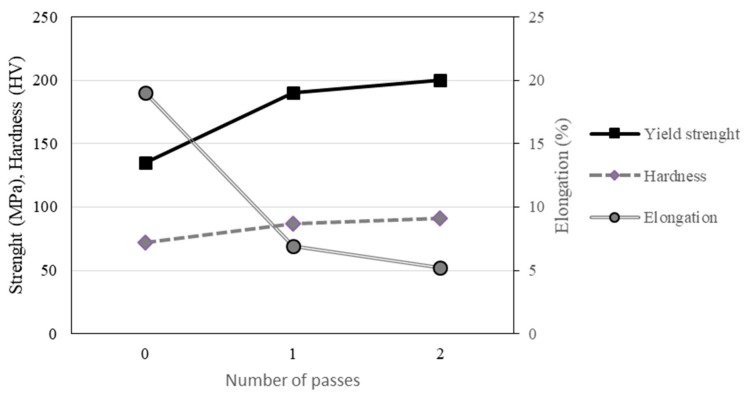
Changes of mechanical properties on the Al sheets as a result of the number of RCS passes.

As expected, with the deformation induced by the RCS process, the yield strength of the Al sheets increased at the expense of a loss in ductility (Figure 4). The most significant changes were found after the first RCS pass.

## 8. Forming Limit Curves

**Figure 5:**
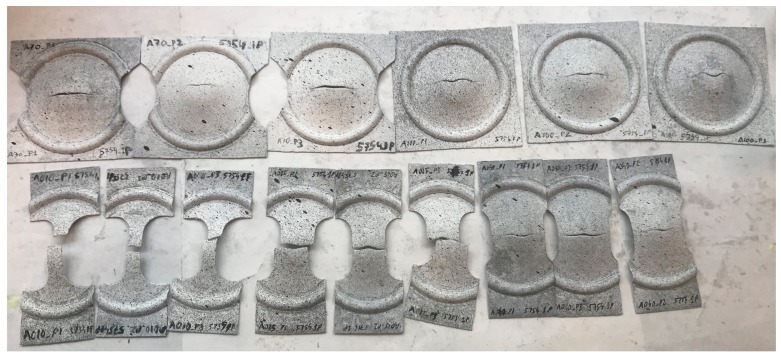
Fractured Nakajima samples for the sheets processed after one RCS pass.

In order to trace the FLC, the minor and major strains were calculated at fracture for the different analyzed geometries. To exemplify, some fractured samples are shown in Figure 5, here the fractures on the different Nakajima geometries can be observed. The fracture always occurred in the center of the specimen, which indicated the repeatability of the tests.

**Figure 6:**
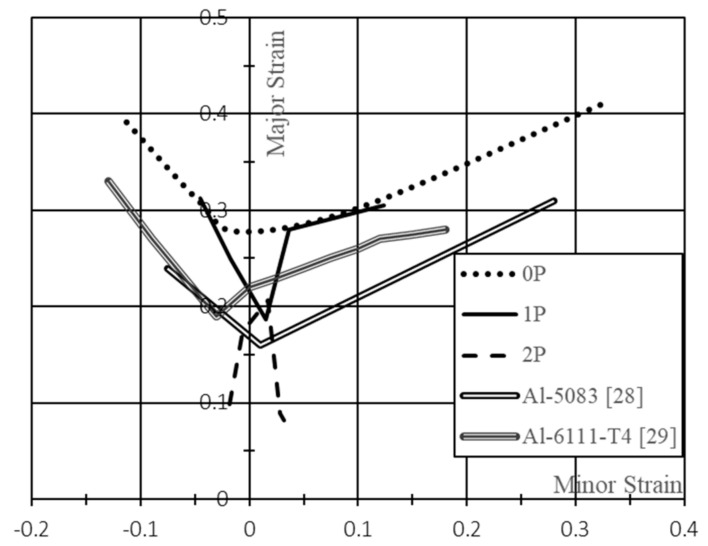
Forming limit curves of the material before and after being processed by RCS, and comparison curves from the literature [28,29].

The FLC changes through the number of RCS passes are shown in Figure 6. The as-received Al sheets showed high strain values at fracture as expected for this kind of material. On the other hand, it can be seen that the strain values significantly dropped as the number of RCS passes increased.

The Al sheets processed after one RCS pass showed a drop in the FLC at minor strain equal to 0, i.e., at plane strain conditions. Nevertheless, for deformation states with minor strains above 0.04, the FLC values of both major and minor strain are overlapped to the Al sheet with no RCS passes. Additionally, minor strains below −0.05 and above 0.12 could not be achieved with the investigated geometries. For the Al sheets with two RCS passes, the deformation states varied significantly, lowering both, the major and minor strain at which the fracture occurred. This behavior indicates a significant loss in the material forming capacity.

**Figure 7:**
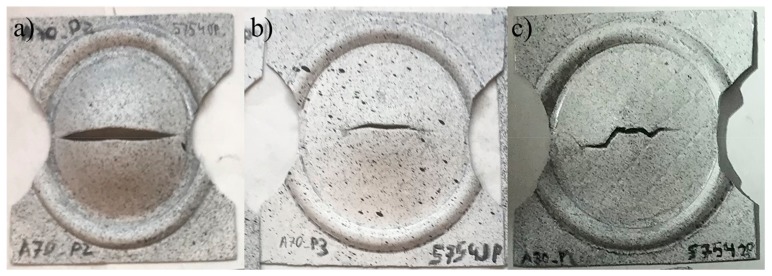
Comparison of the fractures after the Nakajima tests in samples with (**a**) zero (**b**) one and (**c**) two RCS passes.

The profile of the FLC changed even though the same geometries were used. The later could be attributed to the fact that the RCS process induced significant heterogeneous deformation on the Al sheets, changing the usual deformation paths and hence the deformation states. This assumption is supported by the comparison of the fractures for the same sheet geometries with different RCS passes (Figure 7); here, it can be observed that the path followed by the fracture changed drastically from the first to the second RCS pass.

Although there was a loss in forming capacity, the samples with one RCS pass have relatively high forming capacity values when compared to other Al alloys, i.e., AA5083 [28] and AA6111-T4 [29]. The latter is a standard material used in the automotive industry [29].

## 9. Crystallographic Texture

**Figure 8:**
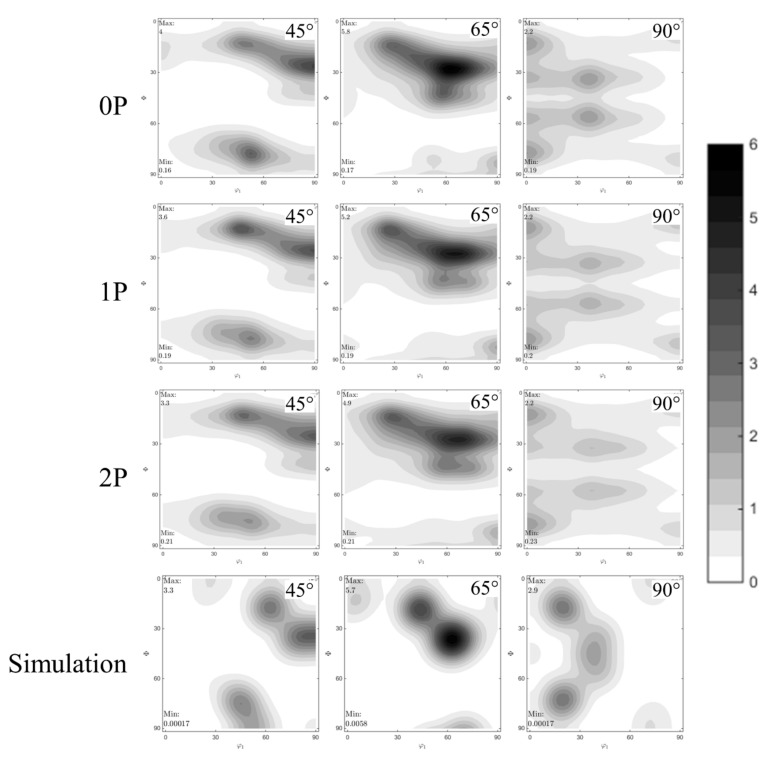
Evolution of the ODF’s on samples at 0P, 1P, 2P deformation passes, and the key components of the identified texture.

The ODF’s of the deformed samples—calculated from (111), (200), and (220) planes—are shown in Figure 8, including the key components of the identified texture. The calculated and measured components (S, Brass, Goss) exhibited a good match. Quantification of the texture fraction volume was carried out in order to track the evolution of the texture components as a function of the number of RCS passes.

The previous observations indicate that the sample had a mixed texture of β-fiber and Goss; no significant cube texture was found, but a fiber connecting S and cube was observed, this is already mentioned as a possible path of recrystallization towards Cube [30]. This fact agrees well with the sample thermomechanical history, as the sample was rolled (generating the β-fiber) and then annealed, producing S- Cube fiber. In order to quantitatively compare the effect of the process on the different texture components, Table 2 lists the volume fraction calculated with MTEX, assuming a unimodal distribution of intensities [27].

From the above, the main texture components observed were S and S-cube fiber, which represents more than 50% of the texture fraction. The texture components remain unchanged during the process. However, the volumetric texture fraction decreased slightly with the number of RCS passes, as shown in Table 2. In addition to the decrement of the β-fiber volume, the fact that the process did not generate more β-fiber could represent a good opportunity to enhance the forming capacity of the material, since it has been reported, for aluminum alloys, that the β-fiber is detrimental for its formability [17,18]. These results confirm that the RCS could induce deformation and mechanical straightening without producing β-fiber, contrary to the rolling process [15,16].

## 10. Strain-Rate Sensitivity

**Figure 9:**
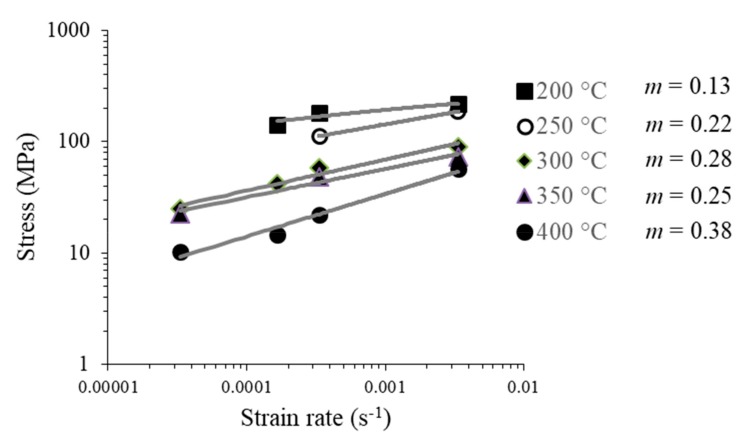
Maximum stress versus strain-rate for different temperatures and their respective *m* coefficient for samples processed by one RCS pass.

The data extracted from the strain-stress curves, for 1P samples, were used for calculating the corresponding strain-rate sensitivity coefficient values. They are plotted in Figure 9. The lowest *m* value was found at 200 °C, increasing as the temperature augmented. The highest *m* value was achieved at 400 °C. These values are in good agreement with the fact that at high temperatures, a new deformation mechanism can be activated [31].

**Figure 10:**
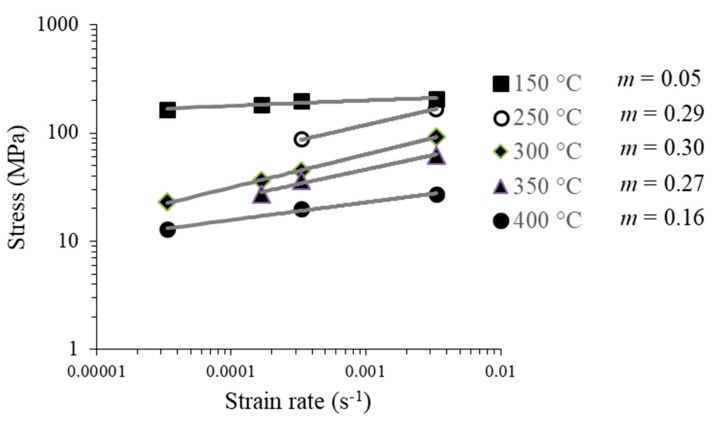
Maximum stress versus strain-rate for different temperatures and their respective *m* coefficient for samples processed by two RCS passes.

For the samples with two RCS passes, there is a similar tendency (Figure 10). In other words, the values of *m* coefficient increased with temperature up to 300 °C. However, at 350 and 400 °C this tendency was modified, and the *m* coefficient dropped to 0.27 and 0.16, respectively. This behavior can be attributed to the possible damage that the Al sheets suffered during the RSC process, being consistent with the FLC results. A similar behavior has also been suggested by [32].

When comparing the “*m*” coefficient of the samples with one and two passes (Figure 9 and Figure 10), it can be seen that at 250, 300 and 350 °C the Al sheets with two passes showed higher strain-rate sensitivity. The Al sheets recrystallization temperature decreased as a result of the RCS process, impacting on the activation temperature associated with the deformation mechanism. The magnitudes of the *m* values are relatively higher than those reported for this alloy, being *m* ≤ 0.05 even for samples deformed [9,31]. This implies that the RCS enhanced the deformation capacity of the alloy at high temperatures.

## 11. Conclusions

The reported new design of repetitive corrugation and straightening process increased the mechanical strength of 5754-aluminum alloy sheets with a reasonable reduction in ductility. The forming limit curves measured the changes in their forming capacity. The samples with one RCS pass showed higher formability values when compared to other commercial Al alloys. The initial texture of the Al samples before being processed by RCS changed slightly in terms of volume fraction, reducing all initial texture components, including β-fiber. The mechanical behavior of the samples was measured at different strain rates and temperatures. The strain-rate sensitivity coefficient increased in comparison to that reported in the literature, showing good forming capacity at relatively high temperature conditions.

## Figures and Tables

**Table 1:** Nominal composition of the 5754-aluminum alloy investigated.

Si	Fe	Cu	Mn	Mg	Cr	Zn	Ti	Al
0.04	0.17	0.00	0.01	3.00	0.22	0.00	0.02	Rest

**Table 2:** Changes of the texturized volume for the components Goss, brass, S, Cu, cube, and the S-cube connecting fiber.

Texture Component	0P (%)	1P (%)	2P (%)
Goss	2.3	2.0	1.7
Brass	6.6	5.1	3.9
S	25.9	21.7	21.7
Cu	6.3	5.7	6.0
Cube	0.7	0.9	0.7
S-cube connecting fiber	18.3	18.3	17.9
